# Tuberculosis: an ongoing global epidemic

**DOI:** 10.1016/j.eclinm.2021.100785

**Published:** 2021-03-26

With the World Tuberculosis Day, which takes place each year on March 24, we get reminded that ancient diseases remain a substantial medical issue worldwide. On March 24, 1882, Robert Koch discovered the bacteria *Mycobacterium tuberculosis* that causes the disease tuberculosis (TB). Back in the 19th century, TB killed one out of every seven people living in the USA and Europe. In 1982, on the 100th anniversary of the discovery of TB, the World Tuberculosis Day was announced. Since then on this day global campaigns led by WHO and other international and national organisations take place to educate the public about the disease.

TB adversely infects groups of the population that historically have had a limited access to health care based on their race or ethnicity. TB cases are higher in Hispanic or Latino, Black or African American, Native Americans, and Asians; in the USA, 88% of all TB cases occurred in racial and ethnic minority groups as reported in 2019 by the Center for Disease Control and Prevention (CDC). Additionally, comorbidities contribute to these disparities; people with diabetes or HIV infections are more likely to develop TB. There is a strong need to overcome these disparities to fight the disease. The CDC is collaborating with international public health organisations to improve TB screening by using culturally appropriate materials and outreaching local communities to educate and raise awareness.

This year, we commemorate the World Tuberculosis Day when nearly 4000 people worldwide still die each day and nearly 28 000 people contract the disease every day. Overall past global efforts to fight TB put in place since 2000 have saved around 63 million lives; but yet this preventable and curable disease claims so many lives globally. In 2015, WHO announced its “End of TB Strategy” vision with the goal to end the TB epidemic by 2035 worldwide. The aim is to achieve a 95% reduction in TB deaths by 2035 compared with 2015, a 90% reduction in TB incidence rate, and to end the financial burden for TB-affected families by 2035. These goals should be achieved by implementing three main pillars according to the WHO strategy. First, an integrated, patient-centred care and prevention strategy must focus on early diagnosis and treatment and the implementation of preventive treatments—such as vaccination in people with high risk. Second, political commitment is needed to implement bold polices and supportive systems by providing the resources required for care and prevention. This commitment should be aligned with the engagement of public and private care sector to support health-care coverage and further regulatory frameworks. Third, increase efforts need to be put towards research and innovation leading to discoveries in diagnosis and treatment as well as rapid uptake and implementation of new tools and interventions. As measures of success and progress, a decline in the incidence rate from 2% per year in 2015 to 10% per year by 2025 and a decline in the projected case–fatality ratio from 15% in 2015 to 6·5% by 2025 must be achieved.

Checking back in on these goals today, the WHO Global TB Report published in October, 2020, indicated a 35% reduction in the absolute numbers of TB deaths compared with 2015 and a 20% reduction in the TB incidence rate compared with the baseline numbers from 2015. This success experienced great setbacks because of the COVID-19 pandemic. The CDC reports that unless drastic additional measures are put in place cases are estimated to increase worldwide. Modelling by the STOP TB Partnership predicted that the COVID-19 pandemic will add an additional 6·3 million cases of TB during 2020–25 and an additional 1·4 million TB deaths. Lockdowns worldwide led to service disruptions and slowed down TB diagnosis, prevention, and treatments and will set back the fight against TB by 5–8 years. To counteract this gap the efforts to achieve the goals set for 2035 must be intensified and TB services must be maintained and extended, especially in high TB burden countries such as India, Indonesia, the Philippines, Sierra Leone, and South Africa. WHO advised to maximise remote care and support, and with that reduce the number of visits to health services to ensure limited transmission of TB and COVID-19 in health-care facilities, and additionally to provide simultaneous testing for TB and COVID-19. To highlight this consequential delay the theme for the 2021 World Tuberculosis Day is “The Clock is Ticking” which is intended to convey the sense of urgency to act on the commitments to end TB made by global leaders, especially now in the context of the COVID-19 pandemic. *EClinicalMedicine* wants to highlight this urgent need and calls for fast and bold actions especially in high-risk countries to avoid a catastrophic setback and a return of this preventable disease worldwide.

***EClinicalMedicine***

